# Development of a LAMP assay for the rapid visual detection of the emerging tick-borne Songling virus

**DOI:** 10.1186/s13071-024-06552-7

**Published:** 2024-11-01

**Authors:** Zheng Gui, Yuanning Ren, Qiqi Guo, Weiying Yang, Ziyan Liu, Ning Liu, Yunzhi Peng, Yu Liu, Jingfeng Yu, Lichao Sun, Zedong Wang

**Affiliations:** 1 Department of Infectious Diseases, Center of Infectious Diseases and Pathogen Biology, Key Laboratory of Organ Regeneration and Transplantation of the Ministry of Education, The First Hospital of Jilin University, Changchun, Jilin China; 2 Department of Emergency Medicine, The First Hospital of Jilin University, Changchun, Jilin China; 3 School of Basic Medicine, Inner Mongolia Medical University, Hohhot, Inner Mongolia China

**Keywords:** Songling virus (SGLV), Loop-mediated isothermal amplification (LAMP), Tick, Tick-borne virus, Detection

## Abstract

**Background:**

Songling virus (SGLV) within the genus *Orthonairovirus*, family *Nairoviridae*, is an emerging tick-borne virus associated with human febrile illness. However, no rapid detection method for SGLV has been established.

**Methods:**

In this study, four primer sets targeting the nucleocapsid protein gene of SGLV were designed for use in the LAMP assay and evaluated to identify the optimal primer set. Recombinant plasmids were constructed and utilized for assessing the sensitivity of the assay. Tacheng tick virus 1 (TcTV-1)-, Beiji nairovirus (BJNV)-, Yezo virus (YEZV)-, severe fever with thrombocytopenia syndrome virus (SFTSV)-, and tick-borne encephalitis virus (TBEV)-positive tick samples were utilized to assess the specificity. Field-collected ticks were also evaluated as biological specimens to validate the assay.

**Results:**

A SGLV-specific LAMP assay was established with a detection limit of 1 × 10^–2^ copies/μl and could be visually confirmed by a color change from purple to blue in SGLV-positive samples. No cross-reactivity was observed in the detection of TcTV-1, BJNV, YEZV, SFTSV, and TBEV using the LAMP assay. In addition to the detection of the same seven high-copy numbers of SGLV as the SYBR Green quantitative RT-PCR assay within a reduced timeframe, the developed LAMP method also effectively identified an additional sample with a low copy number in the field-collected tick samples.

**Conclusions:**

We successfully developed a sensitive, specific, and cost-effective visual method for the rapid detection of SGLV using the LAMP assay, which can be applied in pathogenesis and epidemiological surveillance studies of SGLV.

**Graphical Abstract:**

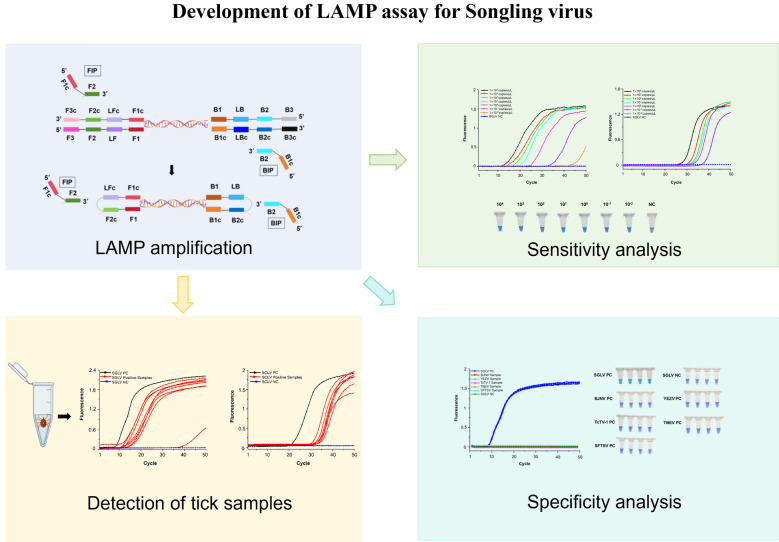

**Supplementary Information:**

The online version contains supplementary material available at 10.1186/s13071-024-06552-7.

## Background

Tick-borne viruses are increasingly emerging on a global scale, posing a significant threat to both human and animal health [1, 2]. Among these viruses, species belonging to the genus *Orthonairovirus* in the family *Nairoviridae*, such as Crimean-Congo hemorrhagic fever virus (CCHFV), Nairobi sheep disease virus (NSDV), Tamdy virus (TAMV), Kasokero virus (KASV), Dugbe virus (DUGV), Yezo virus (YEZV), Tacheng tick borne 1 virus (TcTV-1), and Songling virus (SGLV), can cause mild to severe illnesses and even fatalities in humans and mammals, posing a significant risk to public health security [3–9].

SGLV is an emerging tick-borne nairovirus associated with human febrile illness, which was first identified in humans and ticks in northeastern China [7]. Subsequently, SGLV viral RNA was detected in great gerbils (*Rhombomys opimus*) in northwestern China, indicating the widespread presence of SGLV and underscoring the importance of vigilant surveillance for the virus in endemic areas [10, 11]. Moreover, SGLV infection typically manifests symptoms shared with other tick-borne pathogens, such as Alongshan virus, Yezo virus, Tacheng tick virus 1, and *Rickettsia sibirica*, making it challenging to differentiate the diagnosis based solely on clinical symptoms [12–15]. To date, no rapid detection assays for SGLV have been established. In this study, we investigated and assessed a SGLV-specific LAMP assay based on the nucleocapsid protein gene of the virus, which may offer an efficient and visually accessible detection method.

## Methods

### Tick collection, RNA extraction, and cDNA synthesis

SGLV-, TcTV-1-, BJNV-, YEZV-, SFTSV-, and TBEV-positive tick samples used in this study were kept in the laboratory at − 80 °C. A total of 180 questing *Haemaphysalis concinna* ticks, collected from Inner Mongolia, northeastern China, during April to July 2023, were used to validate the SGLV-specific LAMP assay. Every ten ticks were pooled based on their collection sites and then homogenized using Tissuelyser (Jingxin, Shanghai, China) at 70 Hz for 1 min. After being centrifuged at 12,000 rpm at 4 °C for 10 min, the supernatant from the tick samples was stored at − 80 °C until use. All the tick samples were utilized for RNA extraction employing the TIANamp Virus RNA Kit (Tiangen, China) and reverse transcript to complementary DNA using PrimeScript RT Master Mix (TaKaRa, Japan) following the manufacturer’s protocols.

### Design and screen of LAMP primers for SGLV

Four primer sets targeting conserved sequences of the nucleocapsid protein gene from four SGLV strains with complete genomes available in the GenBank were designed and evaluated to identify the optimal primers (Table S1). The candidate amplification primers, including two outer primers (F3 and B3), one forward inner primer (FIP), one backward inner primer (BIP), one forward loop primer (LF), and one backward loop primer (LB), were designed using the Primer Explorer V5 (https://primerexplorer.jp/lampv5/index.html) and synthesized by Sangon Biotechnology (Table S2). The cycle threshold of the LAMP reaction was determined using the Accurate 96- × 6 Real-Time PCR detection system (Dragonlab). This cycle threshold demonstrated a negative correlation with amplification efficiency, and a primer set exhibiting a low cycle threshold was considered superior.

### Plasmid construction

A 255-bp SGLV fragment containing the amplification region of the designed primers was synthesized based on the sequence of SGLV strain NE-TH1 (GenBank accession no. ON408078). After being digested with *Sma* I, the fragment was cloned into the pUC57 vector and transformed into an *Escherichia coli* competent cell. The positive plasmid was obtained according to blue/white selection and identified by colony PCR. The fragment copies were calculated using the following formula: X copies/μl = [6.02 × 10^23^ (copies/mol) × DNA concentration (g/μl)]/average molecular weight of a base (g/mol) × template length. The cDNA concentration was measured with a UV-Vis spectrophotometer Q5000 (Quawell Technology).

### LAMP reaction

The LAMP amplification system was performed in a final reaction volume of 25 μl, which contained 2.5 μl of 10 × isothermal amplification buffer, 1.5 μl MgSO4 (100 mM), 3.5 μl dNTP Mix (10 mM), 1 μl *Bst* 2.0 WarmStart DNA polymerase (320 U/ml), 1 μl each of FIP and BIP (1.6 μM), 1 μl each of F3 and B3 (0.2 μM), 1 μl each of LF and LB (0.4 μM), 1 μl Eva green, 1 μl hydroxynaphthol blue (HNB), 1 μl cDNA template, and 8.5 μl ultrapure water. Negative controls (ultrapure water) were included in each LAMP reaction. The reaction was carried out within the temperature range of 61 to 66 °C for 50 min. Change of color from purple to blue observed directly by the naked eye was considered SGLV positive. The transition from purple to blue, discernible to the unaided eye, was deemed indicative of a positive SGLV result.

### SYBR Green quantitative RT-PCR reaction

SGLV-specific SYBR Green quantitative RT-PCR (RT-qPCR) was conducted as the comparison method using the primers described in the previous study [7]. Each 20 μl reaction volume consisted of 10 μl TB Green Premix Ex Taq II (TaKaRa, Japan), 0.2 μl each of forward and reverse primers, 1 μl cDNA template, and 8.6 μl ultrapure water. The reaction was conducted with the following parameters: initial denaturation at 95 °C for 30 s followed by annealing at 95 °C for 5 s and extension at 60 °C for 30 s over a total of 50 cycles.

### LAMP sensitivity and specificity analysis

Serial tenfold dilutions of the recombinant plasmids were prepared using ultrapure water, ranging from 10^–2^ to 10^4^ copies/µl, and were employed to evaluate the sensitivity of the SGLV-specific LAMP assay. The detection of each gradient was repeated three times. For specificity analysis, except for SGLV, cDNA from five other tick-borne viruses, including TcTV-1, BJNV, YEZV, SFTSV, and TBEV, were examined for cross-reactivity. The reaction mixtures were incubated at 65 °C for 50 min. For the negative control, the cDNA template was substituted with ultrapure water.

### Detection of SGLV in field-collected tick samples

A total of 18 tick pools were examined using the SGLV-specific LAMP assay and validated against the SYBR Green RT-qPCR as a comparative method. Ultrapure water was employed as the cDNA template in the reaction systems to serve as the negative control.

## Results

### Screen of LAMP primers and optimization of reaction temperature

**Fig. 1:**
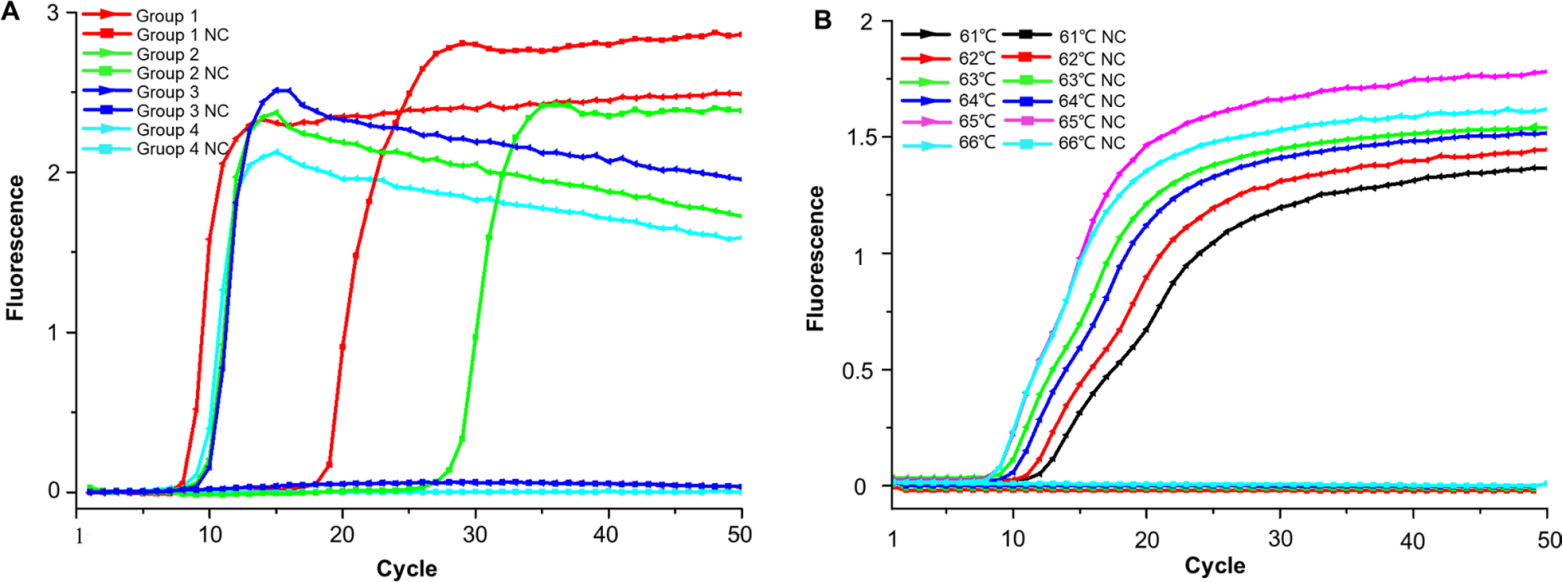
Optimization of LAMP primer sets for Songling virus (SGLV). **A** Real-time fluorescence of different SGLV-LAMP primer groups. **B** Temperature gradient tests (61–66 °C) of the LAMP assay for SGLV

Four pairs of LAMP primers targeted at the nucleocapsid protein gene of SGLV were designed to select the optimum primer set (Table S2). The third primer set demonstrated the highest amplification efficiency for detecting recombinant plasmids at a concentration of 1 × 10^6^ copies/μl (Fig. 1A). The location of the primers used in this study were marked with different colors (Fig. S1). The threshold detection time achieved by the specific primers was 9 min, and no non-specific amplifications were detected in the negative controls. Furthermore, a total of eight positive and eight negative replicates were utilized, resulting in robust outcomes (Fig. S2).

To optimize the amplification signal of the LAMP reaction, we investigated the impact of reaction temperature in the range from 61 to 66 °C. A temperature of 65 °C was found to be optimal for the LAMP reaction in detecting SGLV (Fig. 1B).

### Sensitivity of SGLV-specific LAMP assay

**Fig. 2:**
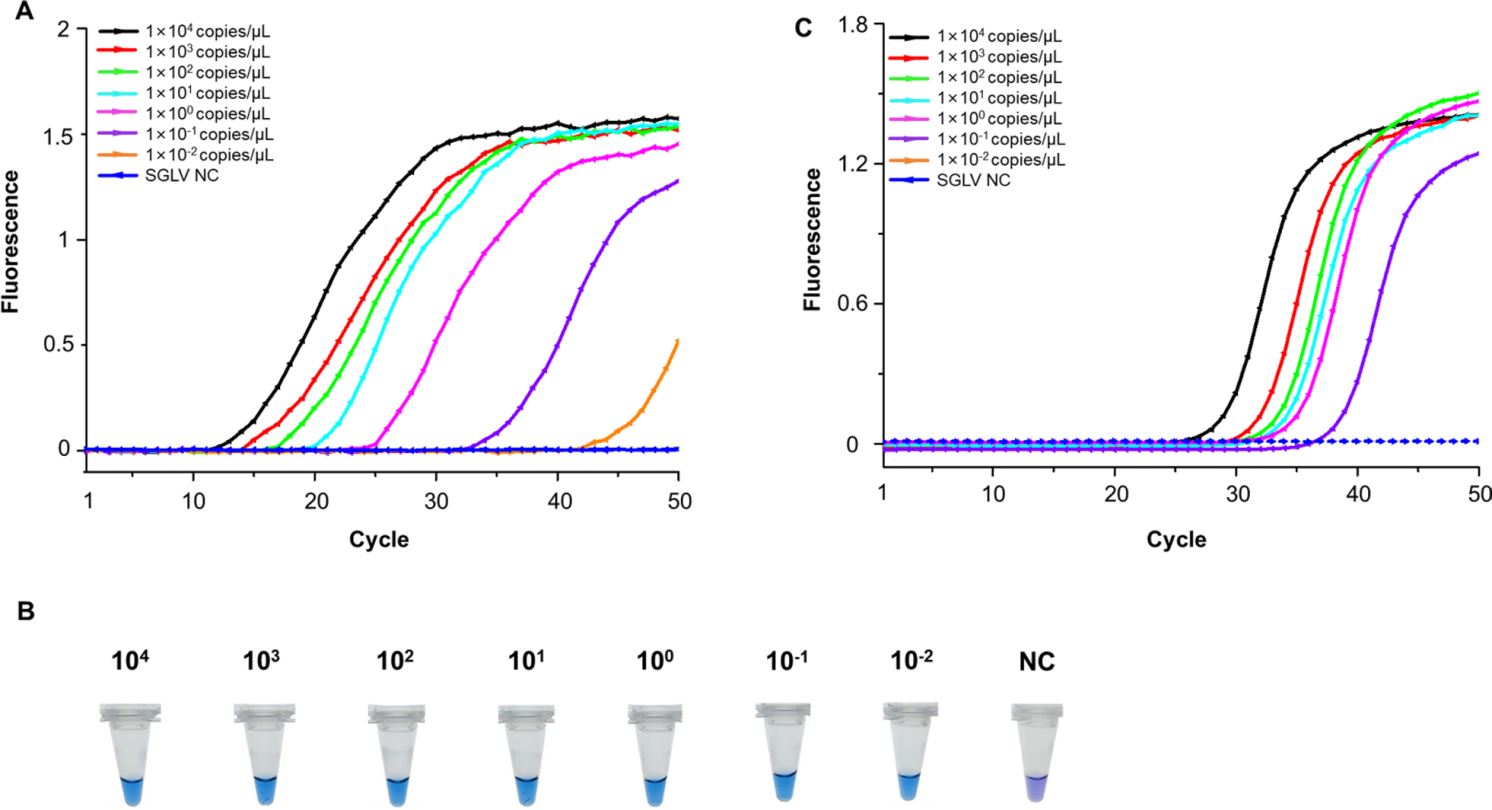
Sensitivity analysis of Songling virus (SGLV)-specific LAMP assay. **A** Sensitivity of SGLV LAMP assay under various concentrations of recombinant plasmids examined using real-time fluorescence. **B** Sensitivity of LAMP assay examined using visible fluorescence method. The plasmids were serially diluted from 1 × 10^4^ copies/μl to 1 × 10^–2^ copies/μl (average of four technical replicates). NC, negative control. **C** Sensitivity of SGLV SYBR RT-qPCR assay under various concentrations of recombinant plasmids examined using real-time fluorescence

The sensitivity of the SGLV-specific LAMP assay was assessed using tenfold serial dilutions of recombinant plasmids. The detection limit was 1 × 10^–2^ copies/μl, with all positive amplifications achieved in under 43 min (Fig. 2A). Except negative control, change of color from purple to blue was observed in all the serially diluted samples directly by the naked eye (Fig. 2B). Meanwhile, the serially diluted recombinant plasmids were also tested by SYBR Green RT-qPCR described in a previous study [7]. The detection limit was 1 × 10^–1^ copies/μl, indicating a sensitivity ten times lower than that of the LAMP assay for SGLV detection (Fig. 2C).

### Specificity of SGLV-specific LAMP assay

**Fig. 3:**
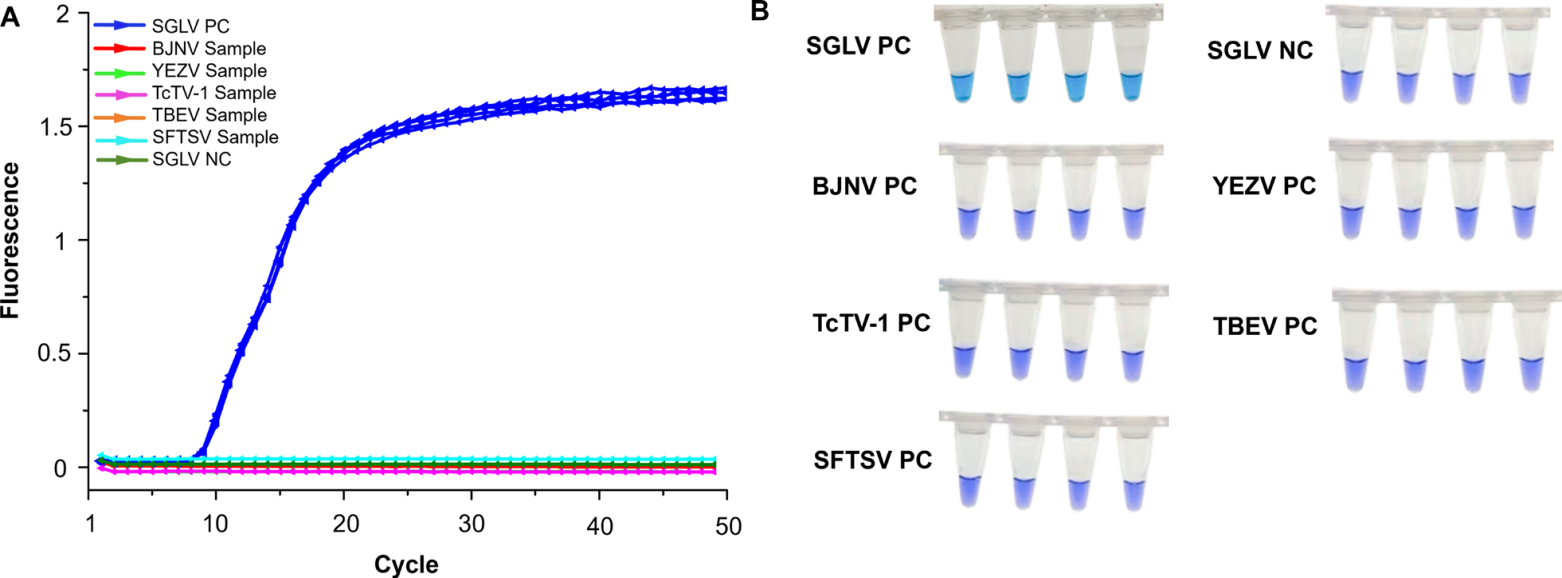
Specificity analysis of Songling virus (SGLV)-specific LAMP assay. **A** Specificity of LAMP assay for the discriminative detection of SGLV and other tick-borne viruses using the selected primer set. **B** Visualization of LAMP reaction products of SGLV and other tick-borne viruses. Beiji nairovirus (BJNV), Yezo virus (YEZV), Tacheng tick virus 1 (TcTV-1), tick-borne encephalitis virus (TBEV), and severe fever with thrombocytopenia syndrome virus (SFTSV). *PC* positive control, *NC* negative control

To evaluate the specificity of the LAMP assay, five tick-borne viruses closely related to or overlapping in epidemic areas with SGLV, namely TcTV-1, BJNV, YEZV, SFTSV, and TBEV, were tested. No specific amplification curves were observed for the five tick-borne viruses, indicating absence of cross-reaction between SGLV and other viruses in the assay (Fig. 3A). Moreover, the tube containing SGLV viral cDNA was confirmed as positive by a color change from purple to blue, while other viruses remained purple, demonstrating the precise identification of SGLV in the LAMP assay (Fig. 3B).

### Detection of SGLV in field-collected ticks

**Fig. 4:**
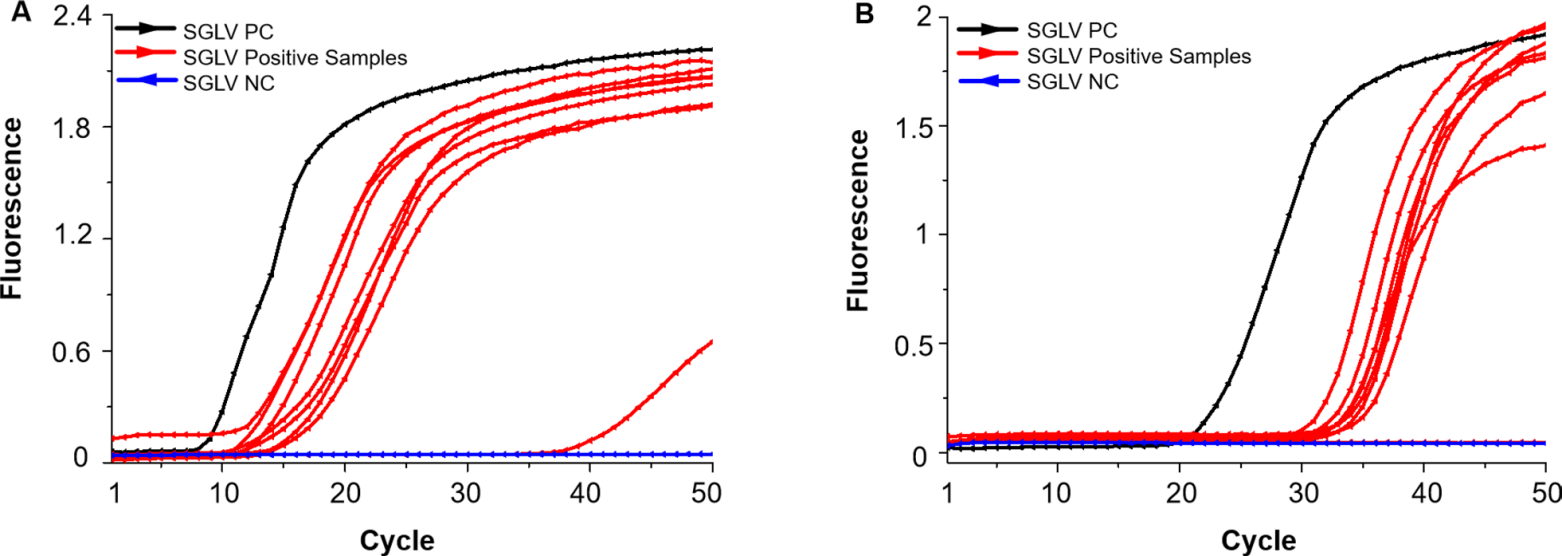
Detection of Songling virus (SGLV) in field-collected ticks from Inner Mongolia, northeastern China.** A** Detection of SGLV in ticks by LAMP assay. **B** Detection of SGLV in ticks by SYBR RT-qPCR assay. *PC* positive control, *NC* negative control

The SGLV-specific LAMP assay was then used to detect 18 pools of field-collected tick samples. The assay showed that eight tick samples were positive for SGLV, with cycles ranging from 12 to 38 (Fig. 4A). The tick samples were also verified using SYBR Green RT-qPCR, and only seven tick samples tested SGLV positive with cycle threshold values between 30 and 32 (Fig. 4B). In addition to facilitating the detection of the same seven high-copy numbers of SGLV as SYBR Green RT-qPCR within a reduced timeframe, the developed LAMP method also successfully identified an additional sample exhibiting a low cycle threshold among the field-collected tick samples (Fig. 4A).

## Discussion

Songling virus is a newly discovered tick-borne orthonairovirus, causing febrile illness in tick-bitten patients in northeastern China [7]. In the previous studies, viral metagenomic analysis, SYBR Green RT-qPCR, semi-nested PCR, indirect enzyme-linked immunosorbent assay (ELISA), and indirect immunofluorescence assay (IFA) have been used for SGLV detection in humans, great gerbils (*Rhombomys opimus*), and ticks [7, 10, 11, 16]. Among these approaches, viral metagenomic analysis is time-consuming and costly, while semi-nested PCR and RT-qPCR require specialized equipment and skilled technicians. Moreover, IFA and ELISA are susceptible to serological cross-reactivity among closely genetically related viruses.

Indeed, timely field detection of viral infections in both humans and animals is important to curb the spread of viruses. However, the previously mentioned detection techniques are inadequate to address the urgent requirements. The LAMP assay is a simple and effective diagnostic technique that requires only a conventional water bath or heat block to amplify template cDNA under isothermal conditions, and the color change can be easily observed by the naked eye. To date, the assay has been widely employed for the detection of a variety of viruses, such as Dengue virus, Zika virus, influenza viruses, and TBEV [17–20], demonstrating the broad range of potential applications for this method. In this study, the developed SGLV-specific LAMP assay demonstrated a superior detection limit and successfully identified an additional SGLV-positive sample in field-collected ticks compared to SYBR Green RT-qPCR, thereby highlighting its enhanced sensitivity (Fig. 2). It is important to note that, due to the absence of SGLV-positive human or animal samples, we have not yet applied the established method for detecting these samples; thus, further validation of this method's applicability in human and animal sample detection is required.

Our study used five tick-borne pathogenic viruses to verify the specificity of the SGLV targeted LAMP assay. Of these viruses, TcTV-1, BJNV, and YEZV are emerging nairoviruses that cause febrile illness in humans in northwestern and northeastern China [6, 21, 22]. Significantly, TcTV-1 exhibits a close genetic relationship with SGLV, sharing a nucleotide sequence identity of > 60% [7]. Furthermore, SFTSV and TBEV represent the two most significant tick-borne viruses in China, capable of inducing hemorrhagic fever and encephalitis, respectively. Studies have revealed the presence of these two viruses in both the northwestern and northeastern regions of China [23–25]. Our assay successfully detected SGLV with no cross-reactivity with the other five tick-borne viruses, indicating the specificity of the established assay.

## Conclusions

In summary, our LAMP assay offers a sensitive, specific, and cost-effective visual method for the detection of SGLV, thereby facilitating on-site testing of the virus. The establishment of this assay is expected to significantly enhance epidemiological surveillance studies of SGLV and will also facilitate pathogenesis research aimed at a deeper understanding of this emerging virus, thereby strengthening efforts in the prevention and control of tick-borne diseases in China.

## Supplementary Information

Additional file 1: Table S1. Nucleotide sequences of Songling virus utilized for the design of specific LAMP primers. Table S2. The Songling virus-specific LAMP primers designed based on the nucleocapsid protein gene. Fig S1. Locations of the designed Songling virus-specific LAMP primer sets based on the conserved fragment of SGLV nucleocapsid protein gene. Fig S2. Songling virus-specific LAMP assay examined using the optimal primer set with amplification curve of 8 repeated.

## Data Availability

No datasets were generated or analysed during the current study.
