# Supplementary material for: Development of a LAMP assay for the rapid visual detection of the emerging tick-borne Songling virus
Source: Parasit Vectors. 2024 Nov 1;17:447. doi: 10.1186/s13071-024-06552-7 (PMC11529016; doi:10.1186/s13071-024-06552-7)
Supplement: Supplementary file 1 — Additional file 1: Table S1. Nucleotide sequences of Songling virus utilized for the design of specific LAMP primers. Table S2. The Songling virus-specific LAMP primers designed based on the nucleocapsid protein gene. Fig S1. Locations of the designed Songling virus-specific LAMP primer sets based on the conserved fragment of SGLV nucleocapsid protein gene. Fig S2. Songling virus-specific LAMP assay examined using the optimal primer set with amplification curve of 8 repeated. [file 13071_2024_6552_MOESM1_ESM.docx]

**Supplementary Material**

**Table S1.** Nucleotide sequences of Songling virus utilized for the design of specific LAMP primers.

| **Number** | **GenBank Accession** | **Strain** | **Host** | **Location** |
| --- | --- | --- | --- | --- |
| 1 | NC079002 | HLJ1202 | Homo sapiens | Lanxi, Heilongjiang, China |
| 2 | ON408081 | NE-TH2 | *Haemaphysalis conicinna* | Tahe, Heilongjiang, China |
| 3 | ON408078 | NE-TH1 | *Haemaphysalis conicinna* | Tahe, Heilongjiang, China |
| 4 | MT328780 | YC585 | *Haemaphysalis longicornis* | Yichun, Heilongjiang, China |

**Table S2.** The Songling virus specific LAMP primers designed based on the nucleocapsid protein gene.

| **Primer No.** | **Primer name** | **Sequence (5**'→**3')** |
| --- | --- | --- |
| 1 | SGLV-FIP-1 | AGGCGCACTCATACACAGGTGCTGAGATTGCCAGGCTCATG |
|  | SGLV-BIP-1 | TGATGGCATCGTCAGGAGAAGCCGTACTCCTTGGACCACTTC |
|  | SGLV-F3-1 | GCCTCGACATCAAGTCTGC |
|  | SGLV -B3-1 | TGGTCAGAAGAAGGAACTGC |
|  | SGLV -LF-1  SGLV -LB-1 | TGTCCTTCAGCCGTTCGTG  TTCGACTGGTTTGAGAAGAACAAG |
| 2 | SGLV-FIP-2 | AGGCGCACTCATACACAGGTGGAGATTGCCAGGCTCATGC |
|  | SGLV-BIP-2 | CGTCAGGAGAAGCTTCGACTGGGGCTTTCGTACTCCTTGGAC |
|  | SGLV-F3-2 | CCTCGACATCAAGTCTGCC |
|  | SGLV-B3-2 | GGTCAGAAGAAGGAACTGCC |
|  | SGLV-LF-2 | TGTCCTTCAGCCGTTCGT |
|  | SGLV-LB-2 | TTTGAGAAGAACAAGGACCACAAG |
| 3 | SGLV-FIP-3 | TCCAGGCGCACTCATACACAGGATTGCCAGGCTCATGCA |
|  | SGLV-BIP-3 | GCATCGTCAGGAGAAGCTTCGAGGCTTTCGTACTCCTTGGAC |
|  | SGLV-F3-3 | GACATCAAGTCTGCCAGCA |
|  | SGLV-B3-3 | GGTCAGAAGAAGGAACTGCC |
|  | SGLV-LF-3 | CATGTCCTTCAGCCGTTCG |
|  | SGLV-LB-3 | CTGGTTTGAGAAGAACAAGGACCA |
| 4 | SGLV-FIP-4 | GGATGTCCAGGCGCACTCATAGGCTCATGCACGAACG |
|  | SGLV-BIP-4 | TGATGGCATCGTCAGGAGAAGCCGTACTCCTTGGACCACTTC |
|  | SGLV-F3-4 | GGGAGAAGGACGCTGAGAT |
|  | SGLV-B3-4 | TGGTCAGAAGAAGGAACTGC |
|  | SGLV-LF-4 | ACAGGTGCCATGTCCTTCAG |
|  | SGLV-LB-4 | TTCGACTGGTTTGAGAAGAACAAG |

**Supplement Figure 1**

**
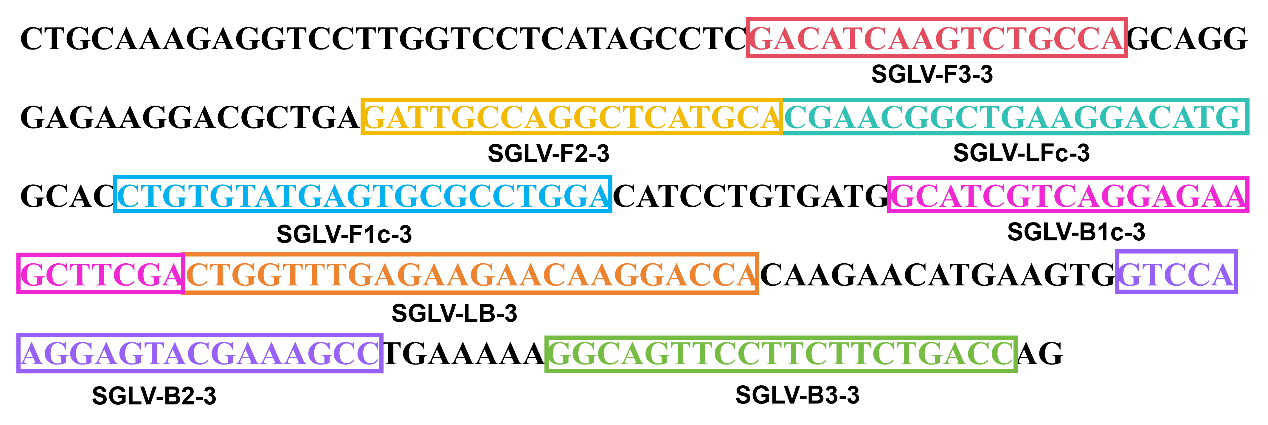
**

**Fig S1. Locations of the designed Songling virus specific LAMP primer sets based on the conserved fragment of SGLV nucleocapsid protein gene.**

**Supplement Figure 2**

**
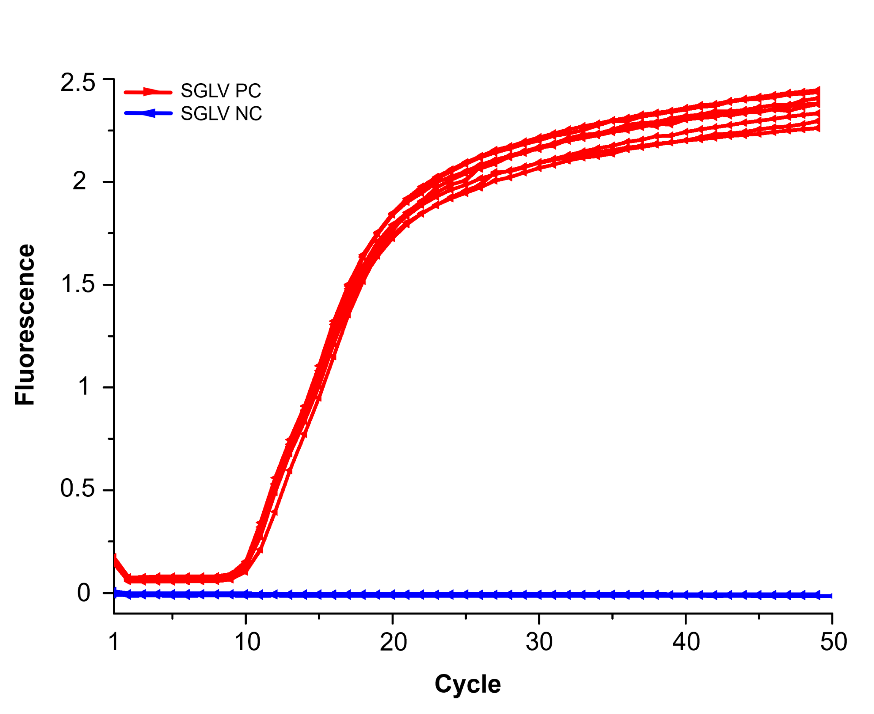
**

**Fig S2. Songling virus specific LAMP assay examined using the optimal primer set with amplification curve of 8 repeated.**
